# Book Reviews

**Published:** 1899-05

**Authors:** 


THE PRINCIPLES AND PRACTICE OF MEDICINE. Designed for the Use of Practitioners and Students of Medicine. By WILLIAM OSLER, M. D., Fellow of the Royal Society; Fellow of the Royal College of Physicians, London; Professor of Medicine in the Johns Hopkins University and Physician-in-Chief of the Johns Hopkins Hospital, Baltimore. Third edition, entirely revised and enlarged. New York: D. Appleton & Co. 1898.

In the March, 1896, number of the JOURNAL appeared a careful review of the second edition of this valuable work and now after a period of three years a third edition is found necessary. The advances made in medicine since the first edition appeared in 1892, has called forth an entire recast of the material, and this edition differs materially from the former editions. Some articles have been entirely rewritten or appear for the first time, such as berriberi, bubonic plague, vaccination, cerebro-spinal fever, malta fever, yellow fever, dengue, influenza, leprosy, glandular fever, gonorrheal infection, gastric neuroses, jaundice, cirrhoses of the liver, diseases of the bile passages, enteroptosis, diseases of the pancreas, thymus, spleen, encephalitis, lymphatism, erythromelalgia and neurasthenia. In the sections on typhoid fever, tuberculosis, rheumatic fever, diabetes, gout, arthritis deformans, parasitic diseases, diseases of the blood, heart, lungs and kidneys much new matter has been incorporated. The chapters on diseases of the nervous system have been rearranged and an attempt has been made to group the diseases in accordance with the modern conceptions of the anatomy and functions of the parts.

The size of the volume is larger somewhat than the preceding editions, the paper heavier and better and a new font of type, furnished by the publishers, thus making it superior in make-up, as well as in contents, to its predecessors.

PROGRESSIVE MEDICINE. A Quarterly Digest of Advances, Discoveries and Improvements in the Medical and Surgical Sciences. Edited by HOBART AMORY HARE, M. D. Professor of Therapeutics and Materia Medica in the Jefferson Medical College of Philadelphia. Philadelphia and New York: Lea Brothers & Co. 1899.

This volume presents the advances in medicine in a most novel and interesting—we had almost said—fascinating manner. It differs very materially from the usual crop of year books that the physician is importuned to purchase, in that it is not a mere collation of material but is a well-digested, interesting narrative of medical progress for a quarter of a year—the design being to issue four volumes annually.

In this first volume are considered questions relating to the surgery of the head, neck and chest; to diseases of children; to pathology; to infectious diseases, including croupous pneumonia; to laryngology and rhinology, and to otology.

The contributors to this volume are: Alexander D. Blackader, J. Chalmers DaCosta, Ludwig Hektoen, Robert L. Randolph, William Sidney Thayer and A. Logan Turner. These will be recognised as men competent to tell the story of medical progress each in his special line—the whole under the editorship of Professor Hobart Amory Hare, one of the most distinguished medical literateurs of the day and one thoroughly competent to judge of the needs of the busy practitioner of medicine.

This first volume is a substantial octavo of nearly 500 pages, printed on heavy book paper, well illustrated and put together in a handsome as well as enduring manner. We predict a large demand for the work, as the yearly price for the set of the four volumes is but ten dollars—a quite nominal sum considering the amount and value of information supplied and the fashion in which it is dressed.

---

A MANUAL OF VENEREAL DISEASES. By JAMES R. HAYDEN, M. D., Chief of Venereal Clinic, College of Physicians and Surgeons, New York; Professor of Genito-urinary and Venereal Diseases in the Medical Department of the University of Vermont, etc. In one 12mo volume of 263 pages, with 47 engravings. Philadelphia and New York: Lea Brothers & Co. 1898.

This the second edition of Hayden's succinct and clear exposition of venereal diseases has come to be looked upon with much favor, as it is but a short time since the first edition was put upon the market. The author has made a general revision of the subject-matter, bringing it down to the very latest researches with the keen acumen of the writer in judging between safe and unsafe measures in treatment; its recommendations, therefore, in regard to therapeutics are valuable and trustworthy.

A volume like the present, affording the student and practitioner a grasp of the best views and methods of this special department, performs a widespread and humane service.

THE PHONENDOSCOPE AND ITS PRACTICAL APPLICATION. By AUREILO BIANCHI, M. D., Professor of Preparatory Clinical Medicine and of Pathology, at Parma, Italy. With 37 illustrations. Translated by A. GEORGE BAKER, A. M., M. D.; Physician-in-chief of the Chinese Medical Dispensary, Philadelphia. Octavo, pp. 77. Philadelphia: George P. Pilling & Son. 1898.

This work is written by the inventor of the phonendoscope, Professor Bianchi. It is well illustrated and will be found helpful to those desiring to acquaint themselves with this instrument and to become expert in its use.

